# Efficiency of Vortioxetine in Depressive Symptoms in Parkinson’s Disease

**DOI:** 10.1192/j.eurpsy.2024.751

**Published:** 2024-08-27

**Authors:** M. Z. Cvitanovic, D. Vukorepa, M. Mustapić, G. Džamonja, M. Čičmir-Vestić, D. Petrić

**Affiliations:** ^1^Department of Psychiatry; ^2^Department of Neurology, University Hospital Split, Split; ^3^Department of Child and Adolescent Psychiatry, Clinical Hospital Centre Rijeka, Rijeka, Croatia

## Abstract

**Introduction:**

Parkinson’s disease (PD) is the most common serious movement disorder in the world, affecting about 1% of adults older than 60 years. The disease is attributed to selective loss of neurons in the substantia nigra, and its cause is enigmatic in most individuals. Patients with PD display both motor and non-motor symptoms. For some patients, the non-motor symptoms are more bothersome than the motor symptoms. One of the most common non-motor symptoms of PD is depression.

**Objectives:**

Treatment of depression with antidepressant drugs is well established. In the last 20 years use of antidepressant has risen mainly due to the introduction of the selective serotonin reuptake inhibitors (SSRIs). Our primary aim was to demonstrate an improvement in depressive symptoms in patients who started treatment with vortioxetine. A secondary aim was to show those who was successfully treated with vortioxetine but was unresponsive to paroxetine and escitalopram without worsening the extrapyramidal symptoms of PD.

**Methods:**

In collaboration with the Department of Neurology, we included patients who are being treated for Parkinson’s disease and who meet the criteria for depressive disorder after a psychiatric examination. We divided the patients into two groups: those who had not previously taken any antidepressant drugs and those who were already on therapy with paroxetine and escitalopram but without the expected therapeutic response. All patients were prescribed vortioxetine in their treatment, and the Hamilton Depression Rating Scale (HDRS) was determined during their first meeting with the psychiatrist, and then again after 6 weeks of taking the medication. Also, we used Mini mental state examination (MMSE) to measure cognitive impairment.Our primary outcome measure was the number of patients in each treatment group who responded to treatment. Response was defined as the proportion of patients who had a reduction of at least 50% from the baseline score on the Hamilton Depression Rating Scale (HDRS)

**Results:**

Our primary outcome measure was the number of patients in each treatment group who responded to treatment. Response was defined as the proportion of patients who had a reduction of at least 50% from the baseline score on the Hamilton Depression Rating Scale (HDRS)

**Conclusions:**

In our research, vortioxetine has proven to be effective in treating depressive symptoms without worsening Parkinson’s disease, unlike paroxetine and escitalopram, which resulted in partial effects.

**Disclosure of Interest:**

None Declared

